# The Big-Five Personality Traits, Maternal Smoking during Pregnancy, and Educational Qualifications as Predictors of Tobacco Use in a Nationally Representative Sample

**DOI:** 10.1371/journal.pone.0145552

**Published:** 2016-01-05

**Authors:** Helen Cheng, Adrian Furnham

**Affiliations:** 1 Research Department of Clinical, Educational and Health Psychology, University College London, London, WC1E 6BT, United Kingdom; 2 ESRC Centre for Learning and Life Chances in Knowledge Economies and Societies, Institute of Education, University College London, London, WC1H 0AL, United Kingdom; 3 BI: Norwegian Business School, Nydalsveien 37, 0484, Oslo, Norway; Children's National Medical Center, Washington, UNITED STATES

## Abstract

**Objective:**

To investigate the associations between the Big-Five personality traits, parental social class, maternal smoking status during pregnancy, childhood cognitive ability, education and occupation, and tobacco use in a longitudinal birth cohort study.

**Method:**

17,415 babies born in Great Britain in 1958 and followed up at 11, 33, and 50 years of age. Lifelong tobacco use status (ever/never) and current tobacco use status (yes/no) at age 50 years were the outcome measures respectively.

**Results:**

Logistic regression analyses showed that among the 5,840 participants with complete data, whilst maternal smoking status, educational qualifications, and all the big-5 personality traits were significant predictors of adult lifelong tobacco use; educational qualifications, own occupational levels, traits Extraversion, Conscientiousness, and Openness were significant predictors of current smoking status. In lifelong measure men tended to have a greater rate of tobacco use than women (52.1% in men and 49.2% in women). However, the sex effect on lifelong tobacco use ceased to be significant once a set of socio-economic and psychological variables in childhood and adulthood were taken into account.

**Conclusion:**

Educational qualifications and the Big-Five personality traits were significantly associated with both current and lifelong tobacco use status.

## Introduction

Tobacco use, both current and lifelong, has been found in much previous research as a major risk factor which has detrimental effects on individuals’ health and lives. Tobacco is the single greatest cause of preventable death globally [1].

There have been a number of studies that have looked at personality trait correlates of health and illness as well as tobacco usage. They have nearly all been cross-sectional, correlational studies that have looked at the relationship between self-reported smoking habits and personality traits. The studies have nearly all used validated self-report personality questionnaires which have related to self-reported smoking history. Most recent studies have used tests that assess the Big Five dimensions of personality which are argued to be orthogonal to each other, relatively stable over time and to a large extent biologically based. They are Neuroticism which assesses emotional sensitivity, instability, and proneness to anxiety and depression; Extraversion which assesses sociability, gregariousness, positive affect and excitement seeking; Openness-to-Experience which assesses curiosity, imaginativeness, and a preference for new experiences; Agreeableness which concerns compassion, empathy, modesty and tender-mindedness; and Conscientiousness which concerns being ambitious, dutiful, reliable and self-disciplined [2].

Whilst the results are inevitably equivocal, all of the Big Five traits have in different studies been shown to relate to various aspects of smoking. Extraverts, Neurotics and those Open-to-Experience tend more to smoke, while those who are Agreeable and Conscientious tend less to smoke.

Most previous research in the area has established Conscientiousness as a protective factor of a number of health conditions [2–3] and as a predictor of longevity [4]. In a meta-analysis Bogg and Roberts [5] quantitatively synthesized 194 studies and found that Conscientiousness-related traits were negatively related to all risky health-related behaviours and positively related to all beneficial health-related behaviours. Combining the results of 46 studies with a total N of 46,725 they found a correlation of r = -.14 between tobacco use and health related behaviours (Q statistic for confidence intervals = 352.83). Correlations between ever-smoked, quality and frequency and health behaviours were very similar. They also showed that two facets of trait Conscientiousness, namely industriousness and self-control were both equally highly correlated with tobacco use (r = -.21).

Looking specifically at smoking status in a meta-analysis based on 25 studies, Munafò and colleagues [6] found that fixed-effects framework indicated a significant difference between smokers and non-smokers on both Extraversion and Neuroticism traits, which remained significant when a random-effects framework was used. The effect-sizes were small and they recommended more longitudinal studies on representative populations. In another study on 1,102 adult Americans, Terracciano and colleagues [7] found that compared to never smokers, current cigarette smokers scored lower on Conscientiousness and higher on Neuroticism. In a recent study, Campbell and colleagues [8] found that smokers had significantly higher qualities of Openness to Experience (*p* < .001) and a lower levels of Conscientiousness (*p* = .07). However, like other studies the sample size was relatively small (n = 69) in the study [7].

Some of these studies have examined personality facets, rather than domain scores [9–10], while others have looked at the personality disorders and smoking [11]. One study looked at personality trait predictors of smoking after the onset of disease, identifying “healthy neuroticism” where Neuroticism reduced smoking after a health scare [12].

In a 10-year study with 2,101 American adults Zvolensky and colleagues [13] found higher levels of Openness and Neuroticism were associated with increased risk of any lifetime cigarette use. Neuroticism also was associated with increased risk of progression from ever-smoking to daily smoking and persistent daily smoking while Conscientiousness was associated with decreased risk of lifetime cigarette use, progression to daily smoking, and smoking persistence. More importantly they showed that these associations between smoking and personality persisted after adjusting for demographic characteristics, depression, anxiety disorders, and substance use problems.

Overall the personality and smoking literature has revealed reasonably consistent findings showing that those high on Extraversion, Openness and Neuroticism, but low on Agreeableness and Conscientiousness tend to have greatest experience of tobacco usage. Whilst many results have been replicated, effect sizes have been modest. More importantly many studies have not taken into consideration other individual difference and demographic factors which could act as moderating or mediating factors, such as intelligence. Through logistic regressions this study looks at the relative effect sizes of personality over other salient variables in explaining smoking behaviour.

Previous studies have demonstrated the associations between childhood intelligence and diseases and mortality [14–15]. The link between socio-economic conditions and health outcomes has been well established [16]. Trait Conscientiousness has also been found to be strongly associated with occupation and career success [17].

Studies in this area have been handicapped either by small and unrepresentative population groups, cross-sectional studies or those which did not include other important participant factors like intelligence or social status. Our aim was to investigate the predictive power of parental social class, maternal smoking as well as an individual’s intelligence, educational achievement and work attainment and personality traits in predicting smoking behaviour at age 50 years. Our particular interest was in personality trait correlates of smoking behaviour.

The current study has two advantages: It used a large, nationally representative birth cohort; and it examined the two main components of individual differences (personally and intelligence) together with a set of social factors in childhood and adulthood.

### Hypotheses

Based on the previous research reviewed above, it was hypothesised 1) Traits (a) Extraversion, (b) Neuroticism, and trait (c) Openness would be positively and trait (d) Conscientiousness negatively associated with tobacco use [2,3,5,15]; 2) Parental social class would be negatively associated with tobacco use [1]; 3) Childhood intelligence would be negatively associated with tobacco use [14,15]; 4) Educational qualifications would be negatively associated with tobacco use [1,16]; 5) Occupational levels would be significantly associated with tobacco use [1,16]. In particular, the study was designed to investigate whether each of the set of inter-correlated socio-economic and psychological factors in childhood and adulthood would explain unique variance of the outcome variables respectively.

## Method

### Sample

The National Child Development Study 1958 is a large-scale longitudinal study of the 17,415 individuals who were born in Great Britain in a week in March 1958 The research team contacted every parent whose child’s birth was registered in that week and invited them to take part in a survey. Information was collected on the family background of the mother, her pregnancy and labour, and about her baby at birth and during its first week of life [18]. There was a follow up seven years later. Since then there have been eight other major surveys, attempting to trace all those born in the week of the original 1958 survey—in 1969, 1974, 1981, 1991, 1999/2000, 2004/5, 2008/9 and most recently in 2013. The following analysis is based on data collected when the study participants were at birth, at ages 11, 33 and at 50 years. Children at age 11 completed tests of cognitive ability (response = 87%). At age 50, 8,532 participants completed a questionnaire on personality traits (response = 69%). Respondents also provided information on educational qualifications at age 33, occupational levels at age 50, and current and lifelong tobacco use measured at 50 years (response = 79%). The analytic sample comprises 5,840 cohort members (51 per cent females) with complete data. Analysis of response bias in the cohort data showed that the achieved adult samples did not differ from their target sample across a number of critical variables (social class, parental education and gender), despite a slight under-representation of the most disadvantaged groups [19].

### Measures

#### Childhood measures

1 Parental social class at birth was measured by the Registrar General’s measure of social class (RGSC). RGSC is defined according to occupational status and the associated education, prestige or lifestyle [20] and is assessed by the current or last held job. Where the father was absent, the social class (RGSC) of the mother was used. RGSC was coded on a six-point scale, from unskilled occupations to professional [21].
2 Mothers of cohort members were interviewed at birth and provided information on maternal smoking status (coded as No = 0, Yes = 1), and also on birth weight and gestational age.
3 Childhood cognitive ability tests [22] consisted of 40 verbal and 40 non-verbal items and were administered at school when cohort members were at age 11 years. Children were tested individually by teachers, who recorded the answers for the tests. For the verbal items, children were presented with an example set of four words that were linked either logically, semantically, or phonologically. For the non-verbal tasks, shapes or symbols were used. The children were then given another set of three words or shapes or symbols with a blank. They were required to select the missing item from a list of five alternatives.

#### Adulthood measures

4 At age 33, participants were asked about their highest academic or vocational qualifications. Responses are coded to the six-point scale of National Vocational Qualifications levels (NVQ) which ranges from ‘none’ to ‘university degree or higher’/equivalent NVQ 5 or 6. Data on current or last occupation held by cohort members at age 50 were coded according to the Registrar General’s Classification of Occupations (RGSC), described above.
5 Personality traits were assessed by the 50 questions from the International Personality Item Pool (IPIP) [23]. Responses (5-point, from “Strongly Agree” to “Strongly Disagree”) are summed to provide scores on the Big-Five personality traits: Extraversion, Emotional Stability/Neuroticism, Conscientiousness, Agreeableness, and Intellect/Openness.
6 At age 50 cohort members provided information on current smoking status (coded as No = 0, Yes = 1) and lifelong tobacco use status (coded as Never = 0, Ever smoke = 1).

### Statistical Analyses

To investigate the correlates of current and lifelong tobacco use, first ANOVA and *T*-test were used examining the characteristics of the study population. Second, correlation matrix of the variables used in the study were prepared. Third, a series of logistic regression analyses were conducted using lifelong tobacco use and current tobacco use as dependent variables respectively. STATA version 12 was used for these analyses. As the hypotheses were expressed in terms of correlations all results can be seen in Tables and S1 Appendix.

## Results

### Descriptive Analysis

Table 1 shows the characteristics of the study.

**Table 1:** Social and demographic characteristics of the study population and the rate of current and lifelong tobacco use.

	n	%	Rate of current tobacco use %	Rate of lifelong tobacco use %
*Sex*				
Male	2886	49.4	18.3	52.1
Female	2954	50.6	18.7	49.2
*Parental social class at birth*				
Unskilled (V)	428	7.3	25.2	54.0
Partly skilled (IV)	680	11.6	22.1	53.2
Skilled manual (III)	2846	48.7	19.4	51.4
Skilled non-manual (III)	644	11.0	15.4	48.4
Managerial\tech (II)	923	15.8	14.2	48.0
Professional (I)	319	5.5	12.2	46.4
*Educational qualifications at age 33*				
No qualifications	419	7.2	37.5	69.7
CSE 2-5/equivalent NVQ1	657	11.3	28.3	61.9
O Level/equivalent NVQ2	2014	34.5	19.8	53.3
A level/equivalent NVQ 3	907	15.5	15.1	46.6
Higher qualification/equivalent NVQ4	960	16.4	14.4	46.9
University Degree/equivalent NVQ 5, 6	883	15.1	7.1	35.4
*Own current social class at age 50*				
Unskilled (V)	120	2.1	34.2	64.2
Partly skilled (IV)	626	10.7	27.0	57.3
Skilled manual (III)	1026	17.6	25.2	59.8
Skilled non-manual (III)	1213	20.8	17.6	47.0
Managerial\tech (II)	2480	42.5	14.7	47.3
Professional (I)	375	6.4	8.5	43.7

A correlation matrix of the variables examined in the study is shown in S1 Appendix. It shows that current tobacco use and lifelong tobacco use were significantly and positively correlated with trait Extraversion and negatively correlated with Traits Emotional Stability and Conscientiousness. This confirms hypothesis 1a and 1d. A predicted Neuroticism was positively correlated with tobacco use confirming hypothesis 1b, but in contradiction to hypothesis 1c, Openness was unrelated to either of the tobacco use variables.

Parental social class, as well as participant childhood intelligence, education and occupation were all significantly and negatively associated with the two outcome variables. This confirms hypotheses 2, 3, 4 and 5.

### Regression Analyses

Table 2 shows the results of logistic regression analyses using lifelong tobacco use as dependent variable. Model 1 shows that among childhood socio-demographic factors, sex and intelligence were significant predictors of lifelong tobacco use in adulthood. Model 2 shows that after entering adult social factors into the equation, sex, maternal smoking during pregnancy, educational qualifications, and occupation were significant predictors of the outcome variable, and childhood intelligence ceased to be a significant predictor. Model 3 shows that after entering personality factors maternal smoking during pregnancy, educational qualifications, and all the Big-Five personality traits were significant predictors of lifelong tobacco use, and sex was no longer a significant predictor.

**Table 2:** Odds ratios (95% CI) for lifelong tobacco use measured at age 50, according to childhood and adulthood factors.

*Measures*	Model 1	Model 2	Model 3	*p*-value^#^
	Odds ratio (95% CI)	Odds ratio (95% CI)	Odds ratio (95% CI)	
Sex	0.88 (0.79, 0.98)*	0.87 (0.77, 0.98)*	0.88 (0.77, 1.00)	0.056
*Childhood factors*				
Parental social class at birth *(unskilled as reference group)*				
Partly skilled	0.98 (0.76, 1.27)	0.99 (0.76, 1.29)	1.04 (0.79, 1.38)	0.767
Skilled manual	0.97 (0.78, 1.21)	1.06 (0.85, 1.33)	1.08 (0.85, 1.37)	0.526
Skilled non-manual	0.92 (0.70, 1.19)	1.07 (0.82, 1.41)	1.10 (0.83, 1.47)	0.491
Managerial\tech	0.87 (0.67, 1.12)	1.06 (0.82, 1.37)	1.11 (0.85, 1.46)	0.440
Professional	0.87 (0.63, 1.20)	1.22 (0.88, 1.71)	1.24 (0.87, 1.75)	0.229
Maternal smoking during pregnancy	0.92 (0.82, 1.04)	0.87 (0.77, 0.99)*	0.85 (0.74, 0.96)**	<0.010
Childhood intelligence at age 11	0.83 (0.78, 0.87)***	1.01 (0.95, 1.08)	0.98 (0.91, 1.05)	0.500
*Adulthood social factors*				
Educational qualifications *(no qualification as reference group)*				
CSE 2-5/equivalent NVQ1		0.70 (0.53, 0.92)**	0.77 (0.57, 1.04)	0.087
O Level/equivalent NVQ2		0.48 (0.37, 0.61)***	0.51 (0.39, 0.67)***	<0.000
A level/equivalent NVQ 3		0.36 (0.27, 0.47)***	0.37 (0.28, 0.50)***	<0.000
Higher qualification/equivalent NVQ4		0.37 (0.28, 0.49)***	0.38 (0.28, 0.52)***	<0.000
University Degree/equivalent NVQ 5, 6		0.22 (0.16, 0.29)***	0.21 (0.15, 0.29)***	<0.000
Own social class (*unskilled as reference group*)				
Partly skilled		0.76 (0.50, 1.17)	0.79 (0.50, 1.25)	0.318
Skilled manual		0.93 (0.61, 1.41)	0.99 (0.63, 1.54)	0.957
Skilled non-manual		0.61 (0.40, 0.91)*	0.66 (0.42, 1.02)	0.062
Managerial\tech		0.73 (0.49, 1.09)	0.73 (0.47, 1.13)	0.159
Professional		0.78 (0.49, 1.23)	0.80 (0.49, 1.31)	0.384
*Personality factors*				
Extraversion			1.22 (1.14, 1.31)***	<0.000
Emotional stability			0.88 (0.82, 0.93)***	<0.000
Agreeableness			0.92 (0.86, 0.99)*	0.033
Conscientiousness			0.87 (0.82, 0.93)***	<0.000
Openness			1.16 (1.08, 1.25)***	<0.000

*Note*:

*p < .05;

**p < .01;

***p < .001. Controlling for gestational age and birth weight in all three models.

^#^*P*-values of the final model.

This model showed that sex, social class of parents and childhood intelligence were not related to smoking at aged 50 years but that educational qualifications were the most powerful predictors.

Table 3 shows the results of logistic regression analyses using current smoking status as dependent variable. Model 1 shows that among childhood factors, parental social class and intelligence were significant predictors of current smoking status measured at age 50. Model 2 shows that after entering adult social factors into the equation, educational qualifications and occupational levels were significant predictors of the outcome variable, and parental social class and childhood intelligence ceased to be the significant predictors. Model 3 shows that after entering personality factors educational qualifications and occupational levels remained the significant predictors of the outcome variable. In addition, personality traits Extraversion, Consciousness, and Openness were significant predictors of the outcome variable.

**Table 3:** Odds ratios (95% CI) for current tobacco use at age 50, according to childhood and adulthood factors.

*Measures*	Model 1	Model 2	Model 3	*p*-value^#^
	Odds ratio (95% CI)	Odds ratio (95% CI)	Odds ratio (95% CI)	
Sex	1.05 (0.92, 1.20)	1.01 (0.86, 1.17)	1.06 (0.89, 1.26)	0.542
*Childhood factors*				
Parental social class at birth *(unskilled as reference group)*				
Partly skilled	0.87 (0.64, 1.17)	0.88 (0.64, 1.20)	0.91 (0.66, 1.27)	0.593
Skilled manual	0.80 (0.62, 1.03)	0.88 (0.68, 1.15)	0.88 (0.66, 1.15)	0.347
Skilled non-manual	0.68 (0.48, 0.94)*	0.80 (0.57, 1.12)	0.80 (0.57, 1.14)	0.213
Managerial\tech	0.63 (0.46, 0.87)**	0.80 (0.58, 1.10)	0.77 (0.55, 1.18)	0.129
Professional	0.62 (0.40, 0.95)*	0.95 (0.61, 1.47)	0.92 (0.58, 1.47)	0.737
Maternal smoking during pregnancy	1.05 (0.90, 1.22)	0.99 (0.85, 1.16)	0.96 (0.82, 1.13)	0.662
Childhood intelligence at age 11	0.75 (0.70, 0.81)***	0.96 (0.88, 1.04)	0.93 (0.58, 1.01)	0.089
*Adulthood social factors*				
Educational qualifications *(no qualification as reference group)*				
CSE 2-5/equivalent NVQ1		0.75 (0.57, 0.99)*	0.71 (0.53, 0.95)*	0.021
O Level/equivalent NVQ2		0.47 (0.37, 0.60)***	0.45 (0.34, 0.58)***	<0.000
A level/equivalent NVQ 3		0.39 (0.29, 0.52)***	0.36 (0.26, 0.49)***	<0.000
Higher qualification/equivalent NVQ4		0.40 (0.29, 0.54)***	0.36 (0.26, 0.50)***	<0.000
University Degree/equivalent NVQ 5, 6		0.18 (0.12, 0.27)***	0.16 (0.11, 0.24)***	<0.000
Own social class (*unskilled as reference group*)				
Partly skilled		0.77 (0.50, 1.18)	0.80 (0.50, 1.28)	0.345
Skilled manual		0.78 (0.51, 1.18)	0.81 (0.52, 1.28)	0.373
Skilled non-manual		0.54 (0.36, 0.83)**	0.57 (0.36, 0.90)*	0.017
Managerial\tech		0.58 (0.38, 0.88)**	0.56 (0.36, 0.88)*	0.011
Professional		0.46 (0.27, 0.80)**	0.44 (0.25, 0.79)**	0.006
*Personality factors*				
Extraversion			1.27 (1.16, 1.38)***	<0.000
Emotional Stability			0.95 (0.88, 1.03)	0.202
Agreeableness			0.93 (0.85, 1.02)	0.148
Conscientiousness			0.91 (0.84, 0.98)*	<0.019
Openness			1.18 (1.08, 1.30)***	<0.000

*Note*:

*p < .05;

**p < .01;

***p < .001. Controlling for gestational age and birth weight in all three models.

^#^*P*-values of the final model.

The results of Model 3, including all factors for current smoking status were similar to those for lifelong use except personality played a more, and social class a less important role in lifelong use compared to current use. Overall it was clear that educational attainment was the most powerful predictor of tobacco use in this sample.

## Discussion

The results of the current study tended to confirm much of the previous literature and stresses the role of personality trait factors in starting to, and continuing to smoke tobacco. Fifty year old smokers tended to be from lower social class backgrounds, and with less intelligence, educational qualifications and lower occupational status. They also tended to be Neurotic Extraverts (Choleric types), low on Conscientiousness. The results for current and lifelong tobacco use showed similar but not identical patterns.

The current study confirmed the significant associations between tobacco use and traits Extraversion, Neuroticism, Conscientiousness, and Openness. The study extended the literature by showing that lifelong tobacco use was also significantly associated with trait Agreeableness. Moreover, after taking account the effects of parental social class, childhood intelligence, maternal smoking during pregnancy, education and occupation, all the Big-Five personality traits remained the significant predictors of lifelong tobacco use in adulthood. Further, Extraversion, Conscientiousness, and Openness were significant predictors of current tobacco use as well. This confirms many studies in the area which had smaller samples [7, 8]. However we replicated the importance of Conscientiousness as the most important predictor of tobacco use as noted by large meta-analyses [5].

Of all the personality factors Extraversion was the strongest predictor of both variables. This confirms most, but not all other studies [7, 8, 13]. This may be explained in different ways but most parsimoniously in terms of Eysenck’s arousal theory of extraversion. Eysenck’s cortical arousal theory of Extraversion has been succinctly summarised by Revelle [24]: 1) introverts are more aroused than extraverts; 2) stimulation increases arousal; 3) arousal related to performance is curvilinear; 4) the optimal level of arousal for a task is negatively related to task difficulty; and 5) arousal related to hedonic tone is curvilinear. Assumptions 1, 2, and 5 lead to the prediction that extraverts should seek out more stimulation, a quick way of which to acquire it is to have a “quick nicotine fix”.

The second most powerful trait predictor was Openness which was not the case in many studies but confirms the work on smoking and the personality disorders which suggests it is associated with behavioural disinhibition, novelty seeking and behavioural activation [11]. By definition those more Open to experiences are eager to experiment with tobacco when young which may lead to later addiction.

It should be noted that occupation was not significantly associated with lifelong tobacco use, but was significantly associated with current tobacco use. It seems that occupation has a stronger effect on current tobacco use, possibly because of social forces to give up smoking in some occupations and environments. Individual difference correlates of occupational success are high intelligence, low Neuroticism and high Conscientiousness which is the precise opposite pattern of a smoker.

Both correlation and regression results highlighted the role of educational achievement as the most powerful predictor of tobacco usage. Most sociological studies have shown this and noted that education is related to occupational attainment. Although higher education is less likely to be about tobacco usage it probably involves for most people discussions about health. Furthermore people are likely to encounter role models who do not smoke at higher educational institutions who strongly influence their behaviour.

The study was limited to the data set that was available. It would have been particularly interesting to know if, when with what success the participants had attempted to give up smoking. It would also be interesting to know also the place, time and reported subjective need for tobacco, all of which could help how personality factors influence tobacco usage.

One implication of these findings is helping people try to give up smoking. This may mean with Extraverts encouraging some other activities which satisfy their need for stimulation. Similarly it could involve encouraging people to be more conscientious in their monitoring and regulation of the smoking, as well as trying to encourage those with high openness scores to seek safer, alternative substitutes for tobacco. This suggests that those trying to help or advise smokers quit the habit adapt their suggested treatment regime to the personality profile of the individuals.

## Supporting Information

S1 Appendix (DOCX)
