# Supplementary material for: The Big-Five Personality Traits, Maternal Smoking during Pregnancy, and Educational Qualifications as Predictors of Tobacco Use in a Nationally Representative Sample
Source: PLoS One. 2016 Jan 5;11(1):e0145552. doi: 10.1371/journal.pone.0145552 (PMC4701483; doi:10.1371/journal.pone.0145552)
Supplement: S1 Appendix — (DOCX) [file pone.0145552.s001.docx]

**S1 Appendix.** Correlations of the variables used in the study.

|  | *Variables* | Mean (SD) | 1 | 2 | 3 | 4 | 5 | 6 | 7 | 8 | 9 | 10 | 11 | 12 | 13 |
| --- | --- | --- | --- | --- | --- | --- | --- | --- | --- | --- | --- | --- | --- | --- | --- |
| 1. | Current tobacco use | .18 (.39) | **_** |  |  |  |  |  |  |  |  |  |  |  |  |
| 2. | Lifelong tobacco use | .51 (.50) | **.473** | **_** |  |  |  |  |  |  |  |  |  |  |  |
| 3. | Sex | .51 (.50) | **.001** | **-.034** | _ |  |  |  |  |  |  |  |  |  |  |
| 4. | Parental social class | 3.33 (1.24) | **-.086** | **-.041** | -.020 | _ |  |  |  |  |  |  |  |  |  |
| 5. | Maternal smoking during pregnancy | .30 (.46) | **.028** | **-.007** | .008 | -.109 | _ |  |  |  |  |  |  |  |  |
| 6. | Childhood intelligence | 103.9 (12.9) | **-.126** | **-.100** | .076 | .261 | -.112 | _ |  |  |  |  |  |  |  |
| 7. | Educational qualifications | 2.69 (1.46) | **-.191** | **-.174** | -.082 | .330 | -.123 | .488 | _ |  |  |  |  |  |  |
| 8. | Own occupational levels | 4.11 (1.21) | **-.146** | **-.100** | -.018 | .217 | -.066 | .326 | .459 | _ |  |  |  |  |  |
| 9. | Extraversion α=.73 | 29.44 (6.59) | **.059** | **.057** | .077 | .033 | .025 | .022 | .073 | .122 | _ |  |  |  |  |
| 10. | Emotional Stability α=.88 | 28.92 (7.04) | **-.035** | **-.064** | -.075 | -.032 | -.021 | .090 | .087 | .078 | .212 | _ |  |  |  |
| 11. | Agreeableness α=.81 | 36.83 (5.25) | **-.013** | **-.035** | .398 | .044 | .008 | .120 | .086 | .105 | .359 | -.057 | _ |  |  |
| 12. | Conscientiousness α=.77 | 33.98 (5.28) | **-.040** | **-.075** | .104 | .017 | -.010 | .032 | .062 | .089 | .144 | -.178 | .275 | _ |  |
| 13. | Openness α=.79 | 32.55 (5.17) | **-.010** | **.003** | -.016 | .142 | .001 | .280 | .322 | 243 | .397 | -.092 | .345 | .223 | _ |

*Note:* Variables were scored such that a higher score indicated being female, a higher rate on current and lifelong tobacco use, a more professional occupation for parents, higher scores on childhood intelligence, maternal smoking during pregnancy, highest educational qualification, more professional occupation, higher scores on traits Extraversion, Emotional Stability, Agreeableness, Conscientiousness, and Openness. Correlations between the outcome variables and a set of other variables examined are in bold.
